# *Perilla frutescens* Leaf Extract Inhibits Mite Major Allergen Der p 2-induced Gene Expression of Pro-Allergic and Pro-Inflammatory Cytokines in Human Bronchial Epithelial Cell BEAS-2B

**DOI:** 10.1371/journal.pone.0077458

**Published:** 2013-10-18

**Authors:** Jer-Yuh Liu, Yi-Ching Chen, Chun-Hsiang Lin, Shao-Hsuan Kao

**Affiliations:** 1 Graduate Institute of Cancer Biology, China Medical University, Taichung, Taiwan; 2 Center for Molecular Medicine, China Medical University Hospital, Taichung, Taiwan; 3 Institute of Biochemistry and Biotechnology, Chung Shan Medical University, Taichung, Taiwan; 4 Clinical Laboratory, Chung Shan Medical University Hospital, Taichung, Taiwan; Midwestern University, United States of America

## Abstract

*Perilla frutescens* has been used in traditional medicine for respiratory diseases due to its anti-bacterial and anti-inflammatory activity. This study aimed to investigate effects of *Perilla frutescens* leaf extract (PFE) on expression of pro-allergic and pro-inflammatory cytokines in airway epithelial cells exposed to mite major allergen Der p 2 (DP2) and the underlying mechanisms. Our results showed that PFE up to 100 µg/mL had no cytotoxic effect on human bronchial epithelial cell BEAS-2B. Further investigations revealed that PFE dose-dependently diminished mRNA expression of pro-allergic cytokine IL-4, IL-5, IL-13 and GM-CSF, as well as pro-inflammatory cytokine IL-6, IL-8 and MCP-1 in BEAS-2B cells treated with DP2. In parallel to mRNA, the DP-2-elevated levels of the tested cytokines were decreased. Further investigation showed that DP2-indued phosphorylation of p38 MAPK (P38) and JNK, but not Erk1/2, was also suppressed by PFE. In addition, PFE elevated cytosolic IκBα level and decreased nuclear NF-κB level in DP2-stimulated BEAS-2B cells. Taken together, these findings revealed that PFE significantly diminished both mRNA expression and protein levels of pro-allergic and pro-inflammatory cytokines in response to DP2 through inhibition of P38/JNK and NK-κB activation. These findings suggest that PFE should be beneficial to alleviate both allergic and inflammatory responses on airway epithelium in response to aeroallergens.

## Introduction

*Perilla frutescens* leaf in a common garnish and has been widely used as medicinal herb such as “Zisu” in traditional Chinese medicine and “saiboku-to” in Japanese herbal formula for asthma treatment. Previous studies have shown that *Perilla frutescens* leaf extracts possess different biological activities, including inhibition of tumor necrosis factor-α (TNF-α) [1], suppression of IgA nephropathy [2], and anti-inflammatory and anti-allergic activity [3], [4]. Airway epithelium has been considered as a major player not only in physical resistance against pathogen invasion and allergens, but also in activation of immune responses [5], [6]. A series of pro-inflammatory cytokines and chemokines are produced by airway epithelial cells upon stimulation with pathogens and allergens, including IL-6, IL-8, granulocyte macrophage-colony stimulating factor (GM-CSF) and monocyte chemotactic protein (MCP)-1 [7]–[9]. Although *Perilla frutescens* has been reported to possess anti-inflammatory activity and traditionally used for respiratory disorders, its effects on allergen-stimulated airway epithelium and the underlying mechanisms remain sketchy.

House dust mite (HDM) is a major causative factor for airway hypersensitiveness and asthma [10]. Of mite-sensitive individuals, approximately 90% generates IgE antibody responses to well-identified HDM allergens that are categorized into 24-kd group 1 and the 14-kd group 2 allergens like Der p 2 (DP2, derived from *D. pteronyssinus*) on basis of IgE affinity [11], [12]. DP2 is known to trigger both pro-inflammatory and pro-allergic responses on respiratory epithelial cells [8]. In addition to induction of pro-allergic cytokines, it is reported that respiratory cells exposed to DP2 result in up-regulated secretion of pro-inflammatory cytokines and expression of intercellular adhesion molecule-1 [8].

Nuclear factor-kappaB (NF-κB) is a widely distributed transcription factor which is normally sequestered in the cytoplasm as an inactive multi-unit complex bound by an inhibitory protein (Iκ-B) [13]. A number of stimuli can activate the complex through phosphorylation and degradation of Iκ-B, leading to translocation of the active dimer into the nucleus. Nuclear NF-κB binds to the promoter region of genes containing the NF-κB motif and regulates their expressions. The upregulation of NF-κB requires the activation of signaling kinases including mitogen-activated protein kinases (MAPKs) such as p42/p44 extracellular signal-regulated kinase (Erk1/2), c-Jun N-terminal kinase (JNK) and p38 MAPK (P38) for nuclear translocation of NF-κB [14], [15].

The present study was aimed to investigate whether extract of *Perilla frutescens* leaf alleviates DP2-induced pro-inflammatory and pro-allergic responses with emphasis on mRNA expression and production of cytokine and cellular signaling. Non-tumorigenic human bronchial epithelial cell BEAS-2B was used as cell model. Cytotoxicity of DP2 was determined by MTT assay. mRNA expression was analyzed by both RT-PCR and real-time quantitative PCR (qPCR). Kinase activation, cytosolic level of IκBα and nuclear-cytosolic distribution of NF-κB was demonstrated by subcellular fractionation and immunoblotting.

## Materials and Methods

All the experiments were performed by using cell model and there is no animals sacrificed for this study.

### Reagents

[3-(4,5-dimethylthiazol-2-yl)-2,5-diphenyl-tetrazolium bromide (MTT), penicillin, streptomycin and general chemicals were purchased from Sigma-Aldrich (St. Louis, MO, USA). Dulbecco's modified Eagle's medium (DMEM), fetal bovine serum (FBS) and trypsin-EDTA were purchase from Gibco BRL (Gaithersburg, MD). Antibodies against phosphorylated-Erk1/2 (p-Erk1/2), total Erk1/2(t-Erk1/2), phosphorylated-JNK (p-JNK), total JNK (t-JNK), phosphorylated-P38 (p-P38), total P38 (t-P38), IkBα and NF-κB(p65) were purchased from Cell Signaling Technologies (Beverly, MA, USA). Antibodies against glyceraldehyde 3-phosphate dehydrogenase (GAPDH) were obtained from Sigma. HRP-conjugated secondary antibodies against mouse IgG and rabbit IgG were purchased from Abcam Inc. (Cambridge, UK). The transformed human bronchial epithelial cell BEAS-2B was obtained from American Type Culture Collection (ATCC; Rockville, MD, USA).

### Preparation of PFE and determination of total phenolic contents

*Perilla frutescens* plants were purchased from a certificated herbal pharmacy (Chung-Yi Chinese herbal medicine pharmacy, Taichung, Taiwan). After dehydration, 100 g of the *Perilla frutescens* leaf was homogenized into powder and passed through a mesh (0.05 mm). The filtered powder was resuspended into 1 L 100% methanol and shaken at room temperature for 24 hours (h). After filtrating by Whatman No. 1 filter paper, the solution was lyophilized. Stock solution (20 mg/mL) of the extract (PFE) was prepared in DMSO, and stored at -20°C until use for treatment.

Total phenolic constituents of PFE were determined by using the Folin–Ciocalteu reagent incoporating with gallic acid as standard [16]. Briefly, 100 µL sample solution containing 1 mg PFE was added into 46 mL distilled water, and then mixed with 1 mL Folin-Ciocaleu reagent by gently shaking thoroughly. 3 mL 2% Na_2_CO_3_ was added to the mixture and reacted for 2 h with intermittent shaking. The same procedure was repeated to all standard gallic acid solutions (0 – 1000 µg/mL). Absorbance at 760 nm of standards and samples was determined for standard curve and phenolics contents. The analysis revealed that about 24.7±1.16% total phenolics as comparing to gallic acid standard.

### Expression and purification of recombinant DP2

Recombinant DP2 was generated as a recombinant polypeptide with a N-terminal glutathione S-transferase (GST) tag and purified as previously described [17]. Briefly, the *E. coli* BL-21 (Novagen, Madison, WI, USA) strain containing pGEX4T1-DP2 or pGEX4T1 plasmid was used for expression and purification of recombinant DP2 protein and GST control protein, respectively. Expression of recombinant protein was induced with 0.1 mM isopropyl β-d-thiogalactoside (IPTG) and checked by immunoblot probing with specific antibody against GST-tag (Sigma-Aldrich). Purification of recombinant proteins was achieved using affinity chromatography (glutathione Sepharose 4B) and gel filtration (Superdex 75, Amersham-Pharmacia Biotech AB, Upsala, Sweden). Protein concentration was determined by BCA protein assay kit (Pierce Biotechnology, Rockford, IL, USA)

### Cell culture and experimental treatments

The nontumorigenic human bronchial epithelial cell BEAS-2B (ATCC® CRL-2503™) was obtained from American Type Culture Collection (ATCC; Rockville, MD, USA) and cultured in Dulbecco's modified Eagle's medium (DMEM) containing 10% v/v fetal bovine serum (Gibco BRL, Gaithersburg, MD, USA) and 100 µg/mL penicillin/streptomycin (Sigma, St. Louis, MO) at 37°C in a humidified atmosphere containing 5% CO_2_.

For viability analysis, cells at 4×104 cells/mL density were seeded in 24-well plates, and then treated with serial concentrations of PFE (10, 20, 30, 50, and 100 µg/mL), DP2 alone (20 µg/mL) or DP2 (20 µg/mL) with PFE (50 µg/mL) for 24 h. For gene expression and kinase signaling experiments, cells were seeded in 6-well plates at an initial density of 5×105 cells/mL, starved in serum-free medium for 16 h, pretreated with serial concentrations of PFE (5, 15, 30 and 50 µg/mL) for 1 h and then treated with DP2 (20 µg/mL) for 4 h or 30 min, respectively. DMSO and purified GST was used as negative control and control protein.

### Cell viability analysis

Cell viability was determined by MTT assay. BEAS-2B cells were treated with 10, 20, 30, 50 and 100 µg/mL PFE for 24 h, and then incubated with MTT (0.5 mg/mL) at 37°C for 4 h. The viable cell number was directly proportional to the production of formazan, which was dissolved in isopropanol and determined by measuring the absorbance at 570 nm using a microplate reader (SpectraMAX 360 pc, Molecular Devices, Sunnyvale, CA, USA).

### RNA Extraction, RT-PCR and quantitative Real-Time PCR (qPCR)

Total RNA was isolated and purified from cells with different treatments using the RNeasy kit (Qiagen, Valencia, CA, USA) according to the manufacturer's instructions. The purified RNA was used as a template to generate first-strand cDNA synthesis using RevertAid™ First Strand cDNA Synthesis Kit (Fermentas. Life Sciences, St. Leon-Rot, Germany). The primer sequences used for RT-PCR and qPCR were listed in Table 1. RT-PCR experiments were performed in triplicates for each sample. qPCR was performed using the ABI PRISM 7700 sequence detection system (Applied Biosystems, Foster City, CA). For mRNA quantitation, FastStart Universal SYBR Green Master (Roche Applied Science, Mannheim, Germany) was used for Taqman PCR. The threshold cycle numbers were calculated using the ΔΔCT relative value method and normalized to GAPDH. qPCR experiments were performed in duplicates for each sample. The correct size of the PCR products was confirmed by agarose gel electrophoresis.

**Table 1:** Primers used for RT-PCR and qPCR analysis.

Target gene		RT-PCR	qPCR
IL-4	Forward	5′-cagctcgaacacttt gaa-3′	5′-ccaactgcttccccctctg-3′
	Reverse	5′-tctcacctcccaactgct-3′	5′-tctgttacggtcaactcggtg-3′
IL-5	Forward	5′-ctgaggattcctgttcctgt-3′	5′-ctgcctacgtgtatgccatcc-3
	Reverse	5′-caactttctattatccactc-3′	5′-cattggctatcagcagagttcg-3′
IL-13	Forward	5′-gctcctcaatcctctcct gtt-3′	5′-gaaggctccgctctgcaat-3′
	Reverse	5′-gcaacttcaatagtcagg tcc-3′	5′-tctgggtcttctcgatggca-3′
GM-CSF	Forward	5′-gcatgtgaatgccatccagg-3′	5′-cactgctgctgagatgaatgaaa-3′
	Reverse	5′-gcttgtagtggctggccatc-3′	5′-gtctgtaggcaggtcggctc-3′
IL-6	Forward	5′-atgaactccttctccacaagcgc-3′	5′-gtagtgaggaacaagccagagc-3′
	Reverse	5′-caagagccctcaggctggactg-3′	5′-ggcatttgtggttgggtca-3′
IL-8	Forward	5′-agatattgcacgggagaa-3′	5′-ctcttggcagccttcctgattt-3′
	Reverse	5′-gaaataaaggagaaacca-3′	5′-cgcagtgtggtccactctcaat-3′
MCP-1	Forward	5′-gctcgctcagccagatgcaat-3′	5′-actctcgcctccagcatgaa-3′
	Reverse	5′-tgggttgtggagtgagtgttc-3′	5′-ttgattgcatctggctgagc-3′
GAPDH	Forward	5′-accacagtccatgccatcac-3′	5′-atgcctcctgcaccacca-3′
	Reverse	5′-tccaccaccctgttgcttga-3′	5′-ccatcacgccacagtttcc-3′

### Immunoblot

Cells were washed with normal saline and lysed in a lysis buffer (50 mM Tris-HCl, pH 7.5, 150 mM NaCl, 1% v/v Igepal CA-630, 1 mM sodium fluoride, and 10 µg/ml aprotinin and leupeptin). The cell lysates were incubated on ice for 30 min, and then centrifuged at 20,000 g for 15 min. The supernatants were collected as crude proteins for immunoblotting.

Crude proteins (30 µg per lane) were electrophoresed on a 12.5% SDS-polyacrylamide gel, and subsequently transferred onto a nitrocellulose membrane (Millipore, Bedford, MA, USA). The blotted membrane was blocked with 1% w/v bovine serum albumin (Sigma), and then incubated for 2 h with 1/1500 dilution of the specific primary antibodies. After thoroughly washing, the membrane was reacted with 1/2000 dilution of peroxidase-conjugated secondary antibodies. Development was performed using ECL chemiluminescence reagent (Millipore) and the reacted signals were semi-quantitated by densitometric analysis.

### Subcellular fractionation

Cells were washed with normal saline and incubated with a lysis buffer (10 mM HEPES, pH7.6; containing 15 mM KCl, 2 mM MgCl2, 0.1 mM EDTA, 1 mM dithiothreitol, 0.05% v/v Igepal CA-630 and 1 mM PMSF, 1 mM sodium orthovanadate, 50 mM sodium fluoride, 10 µg/mL leupeptin, and 10 µg/mL aprotinin) for 10 min. After centrifugation at 2,500 g for 10 min at 4°C, the supernatant was transferred into a new eppendroff, further centrifuged at 20,000 g for 15 min at 4°C, and then the resulting supernatant was collected as cytosolic fraction. The pellets containing nuclei were washed with normal saline, incubated with a nuclear buffer (25 mM HEPES, pH7.6, 0.1% v/v Igepal CA-630, 1 M KCl, 0.1 mM EDTA, 1 mM PMSF, 1 mM sodium orthovanadate, 2 mM sodium fluoride, 10 µg/mL leupeptin, and 10 µg/mL aprotinin), and then centrifuged at 10,000 g for 15 min at 4°C. The resulting supernatants were collected as nuclear fraction.

### Analysis of cytokine production by ELISA

For cytokine production analysis, cells were seeded in 6-well plates at an initial density of 5×105 cells/mL, starved in serum-free medium for 16 h, pretreated with serial concentrations of PFE (5, 15, 30 and 50 µg/mL) for 1 h and then treated with DP2 (20 µg/mL) for 24 h. DMSO and purified GST was used as negative control and control protein. The concentration of IL-4, IL-5, IL-6, IL-8, IL-13, GM-CSF and MCP-1 was determined in cell free supernatants using DuoSet ELISA kits (R&D Systems, Abingdon, UK) according to the manufacturer's instructions.

### Statistical analysis

Data were expressed as means ± SEMs of the three independent experiments. Statistical significance analysis was determined by using 1-way ANOVA followed by Dunnett for multiple comparisons with the control. The differences were considered significant for *P*<0.05.

## Results

### Effects of PFE on viability of epithelial cell BEAS-2B

**Figure 1:**
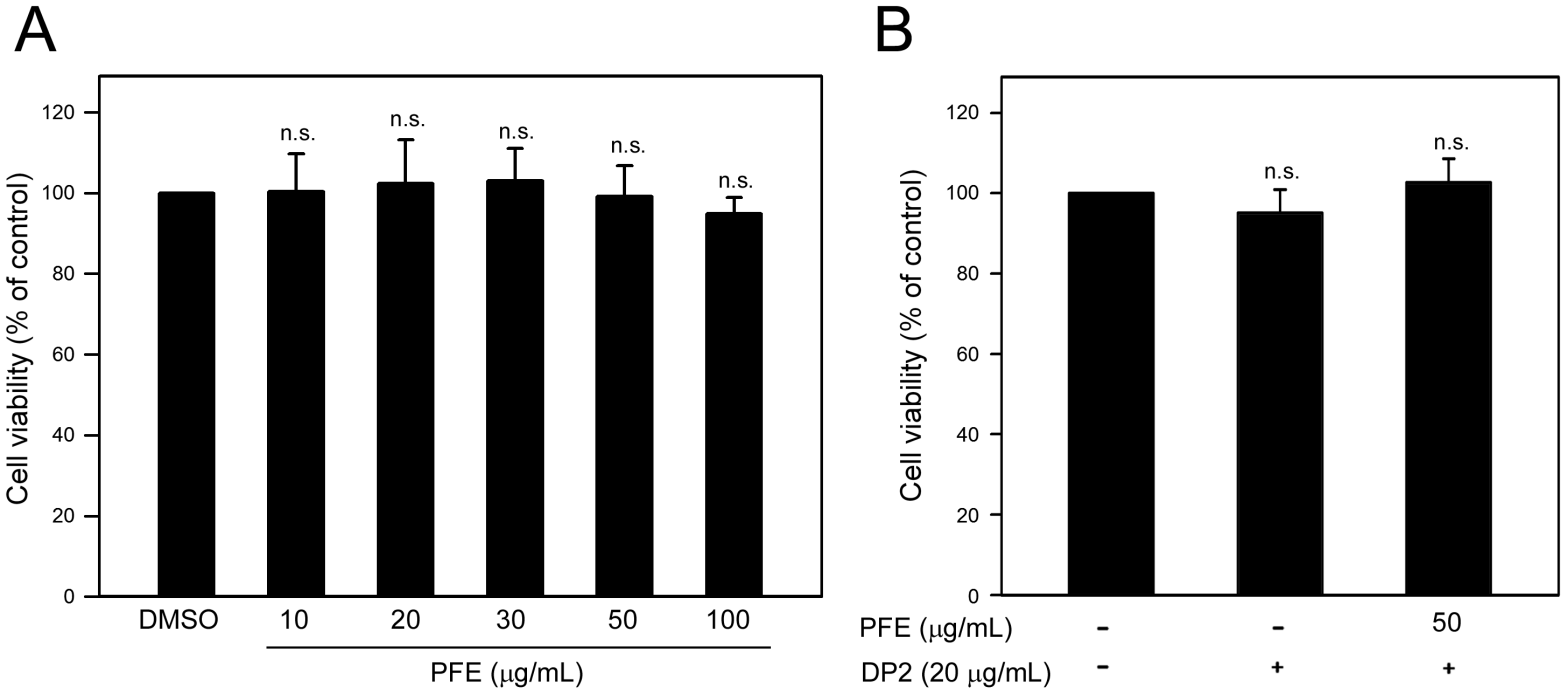
Effects of PFE and DP2 treatment on BEAS-2B cell viability. Cells were cultured in a 24-well plate (4×10^4^ cells/mL) with serum-free medium for 16 h, then (A) treated with serial concentrations of PFE (10, 20, 30, 50 and 100 µg/mL) for 24 h, or (B) pretreated with 50 µg/mL PFE for 1 h and following treated with 20 µg/mL DP2 for 24 h. After the treatments, cell viability was determined by MTT assay. The data were presented as means ± SD of three independent experiments. n.s., not significant as compared to DMSO (A) or control (B).

Cytotoxicity of PFE and DP2 to human airway epithelial cell BEAS-2B was first examined by using MTT assay. As shown in Figure 1A, cell viability of BEAS-2B cells cultured with different concentrations of PFE ranged from 103.4±12.1% to 96.2±9.2% of control, and each changes of viability between DMSO and different concentrations of PFE (10, 20, 30, 50 and 100 µg/mL) were statistically insignificant (*P*>0.127). Based on our previous study [17], 20 µg/mL of DP2 was used to trigger expression of pro-allergic and pro-inflammatory cytokines in BEAS-2B cells, and up to 50 µg/mL of PFE was used for its biological activity investigation. we found that neither DP2 alone (20 µg/mL) nor DP2 combining with PFE treatment (50 µg/mL) significantly affected cell viability of BEAS-2B cells (*P*>0.251 as compared to control)(Figure 1B).

### PFE inhibited DP2-induced mRNA expression and protein production of pro-allergic cytokines in BEAS-2B cells

**Figure 2:**
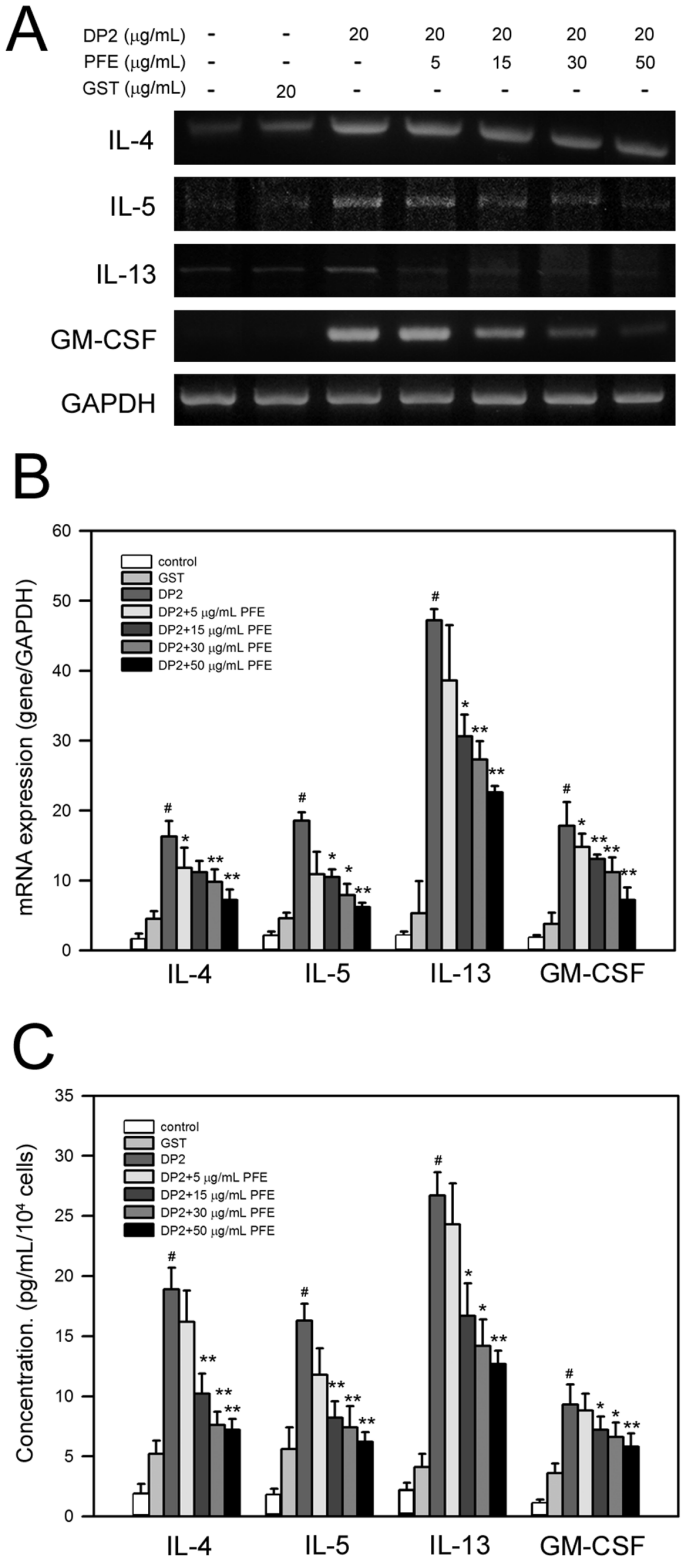
PFE inhibits DP2-induced mRNA expression and protein production of IL-4, IL-5, IL-13 and GM-CSF in BEAS-2B cell. After 16-starvation, cells were pretreated with 5, 15, 30 and 50 µg/mL PFE for 1 h and then stimulated with 20 µg/mL DP2 for 4 h (mRNA expression) or 24 h (protein production). mRNA expression was determined by RT-PCR (A), or by qPCR (B). Protein production was measured by ELISA (C). Quantitative data was shown as means ± SD of three independent experiments. #, *P*<0.05 as compared to GST alone; *, *P*<0.05 and **, *P*<0.005 as compared to DP2 alone.

It is known that DP2 induces secretion of pro-allergic cytokines by respiratory epithelial cells [8]. We next investigated the effects of PFE on mRNA expression of pro-allergic cytokine IL-4, IL-5, IL-13 and GM-CSF in DP2-stimulated BEAS-2B cells. As shown in Figure 2A, significantly induced mRNA expressions of IL-4, IL-5, IL-13 and GM-CSF were observed in DP2-stimulated BEAS-2B cells, and the elevated expressions were inhibited by pretreatment of PFE in a dose-dependent manner. Using qPCR, the results of quantitative analysis revealed that DP2 significantly increased the mRNA levels of IL-4, IL-5, IL-13 and GM-CSF in BEAS-2B cells to 16.3±1.2, 18.5±0.6, 44.1±1.8 and 17.6±2.7-fold of control, respectively (*P*<0.05 as compared to control). GST alone (20 µg/mL) appeared to elevate the mRNA expression of the pro-allergic cytokines, ranging from 3.8±0.9 to 5.1±4.7-fold of control; however, only the increases in IL-4 and IL-5 mRNA expression were significant (*P*<0.05 as compared to control). The DP2-increased mRNA levels of IL-4, IL-5, IL-13 and GM-CSF were reduced by PFE pretreatment in a dose-dependent manner (Figure 2B), and the DP2-upregulated mRNA levels of IL-4, IL-5, IL-13 and GM-CSF were significantly inhibited by PFE pretreatment (50 µg/mL) and decreased to 7.1±0.9, 5.7±0.1, 22.1±1.7 and 7.4±0.8-fold of control, respectively (*p*<0.005 as compared to DP2 alone) (Figure 2B).

In addition to mRNA expression, DP2 increased protein production of IL-4, IL-5, IL-13 and GM-CSF by BEAS-2B cells to 18.5±2.2, 16.7±1.2, 27.5±1.6 and 8.8±1.4 pg/mL/10^4^ cells, respectively (*P*<0.05 as compared to control)(Figure 2C). PFE pretreatment dose-dependently reduced the protein production of the tested cytokines in BEAS-2B cells, and decreased the cytokine production up to 7.6±1.1 (IL-4), 6.6±1.2 (IL-5), 12.4±1.4 and 6.1±1.3 (GM-CSF) pg/mL/10^4^ cells, respectively (*P*<0.05 as compared to DP2 alone)(Figure 2C). Although GST alone (20 µg/mL) elevated the protein production of the pro-allergic cytokines, the increase of the cytokines was insignificant (*P*>0.172 as compared to control).

### PFE inhibited DP2-induced mRNA expression and protein production of pro-inflammatory cytokines in BEAS-2B cells

**Figure 3:**
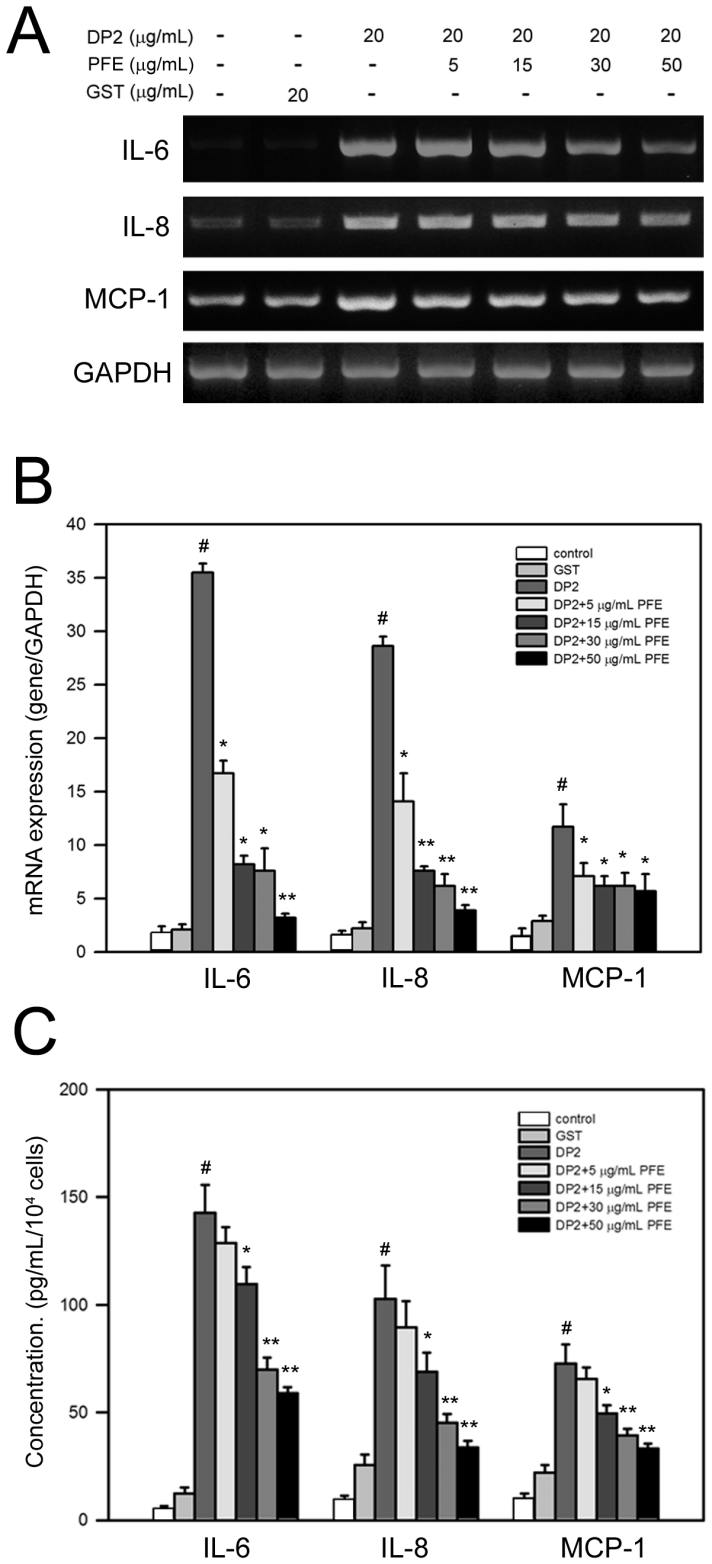
PFE inhibits DP2-induced mRNA expression and protein production of IL-6, IL-8 and MCP-1 in BEAS-2B cell. After 16 h-starvation, cells were pretreated with 5, 15, 30 and 50 µg/mL PFE for 1 h and then stimulated with 20 µg/mL DP2 for 4 h (mRNA expression) or 24 (protein production). mRNA expression was determined by RT-PCR (A), or by qPCR (B). Protein production was measured by ELISA (C). Quantitative data was shown as means ± SD of three independent experiments. #, *P*<0.05 as compared to GST alone; *, *P*<0.05 and **, *P*<0.005 as compared to DP2 alone.

In addition to pro-allergic cytokines, DP2 has been reported to enhance production of pro-inflammatory cytokines by respiratory epithelial cells [8]. We thus investigated whether PFE diminished mRNA expression of pro-inflammatory cytokine IL-6, IL-8 and MCP-1 in BEAS-2B cells in response to DP2. As shown in Figure 3A, DP2 significantly induced mRNA expression of IL-6, IL-8 and MCP-1 in BEAS-2B cells, which was dose-dependently inhibited by PFE pretreatment. Further quantitative qPCR analysis showed that DP2 greatly elevated the mRNA levels of IL-6, IL-8 and MCP-1 in BEAS-2B cells to 35.7±1.6, 28.7±1.1, and 11.3±1.2-fold of control, respectively (*P*<0.05 as compared to control)(Figure 3B). Similar to the results of pro-allergic cytokine expression, GST (20 µg/mL) slightly enhanced the mRNA expression of the pro-inflammatory cytokines, ranging from 1.2±0.4 to 2.1±0.3-fold of control (Figure 3B - 3D). The DP2-elevated mRNA levels of IL-6, IL-8 and MCP-1 were dose-dependently diminished by PFE pretreatment (Figure 3B), and the DP2-elevated mRNA levels of IL-6, IL-8 and MCP-1 were significantly reduced by PFE pretreatment (50 µg/mL) to 2.2±0.3, 1.8±0.2, and 5.9±1.1-fold of control as comparing to control respectively (*p*<0.005 as compared to DP2 alone)(Figure 3B).

In parallel to mRNA expression, protein productions of IL-6, IL-8, and MCP-1 in BEAS-2B cells were increased by DP2 to 142.7±12.9, 102.6±15.6, and 72.7±8.8 pg/mL/10^4^ cells, respectively (*P*<0.05 as compared to control)(Figure 3C). PFE pretreatment diminished the protein production of the tested cytokines by BEAS-2B cells ina dose-dependent manner, and decreased the cytokine production up to 58.9±2.9 (IL-6), 33.7±3.1 (IL-8) and 33.3±2.2 (MCP-1) pg/mL/10^4^ cells, respectively (*P*<0.05 as compared to DP2 alone)(Figure 3C). Additionally, effects of GST alone (20 µg/mL) on the protein production of the pro-inflammatory cytokines examined were insignificant (*P*>0.121 as compared to control).

### PFE inhibited phosphorylation of MAPKs in DP2-stimulated BEAS-2B cells

**Figure 4:**
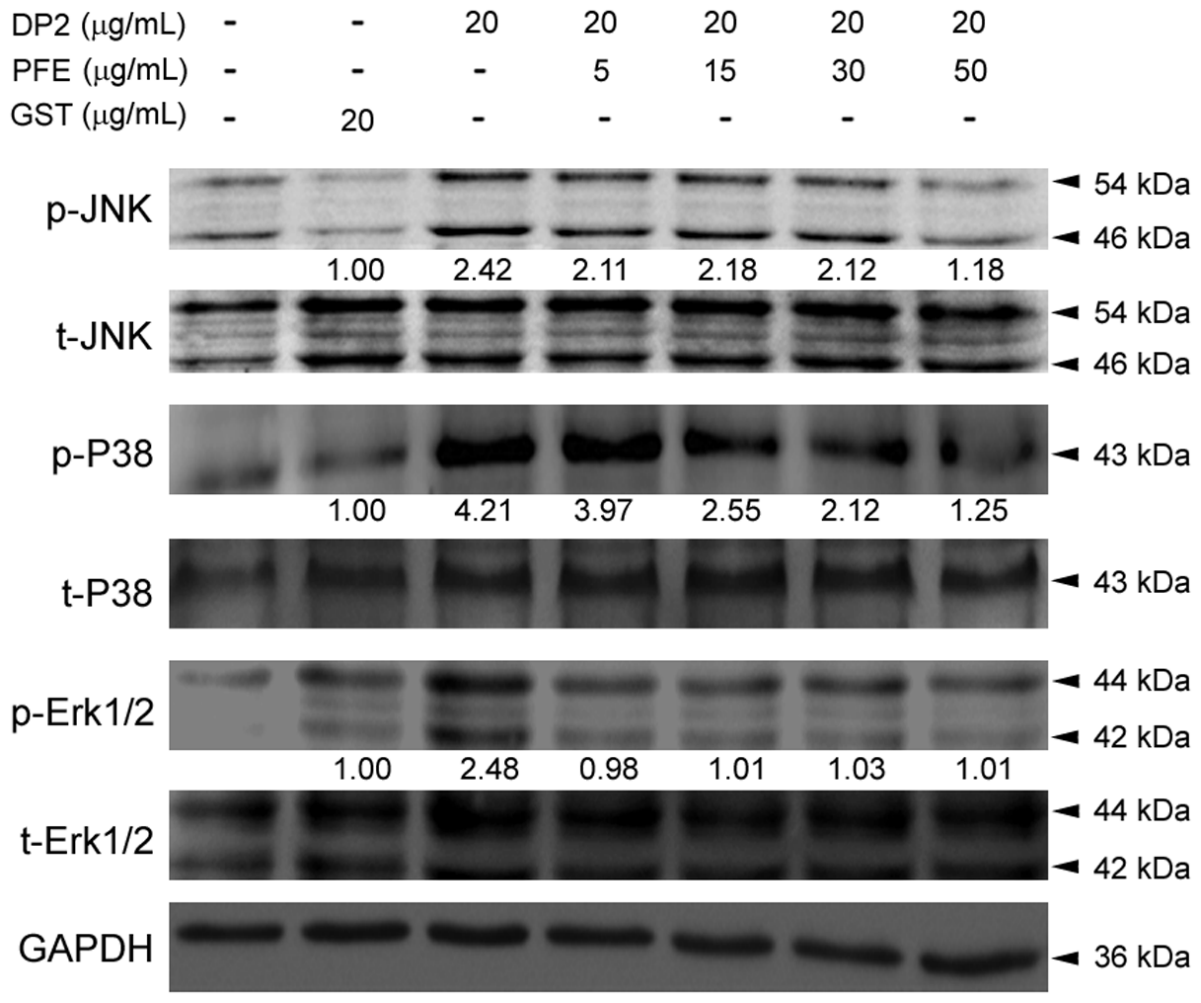
PFE suppressed DP2-induced phosphorylation of MAPKs in BEAS-2B cells. After 16 h-starvation, cells were pretreated with 5, 15, 30 and 50 µg/mL PFE for 1 h and then stimulated with 20 µg/mL DP2 for 30 min. The treated cells were lysed for analysis of phosphorylation by immunoblot using specific antibodies and chemiluminescence development. Quantitative data was performed by densitometric analysis and indicated as [phosphorylated protein/ total protein]. The ratios of GST treatments were used as internal control.

Activation of MAPKs has been known to be associated with DP2-induced production of pro-allergic and pro-inflammatory cytokines [8], [18]. Therefore, effects of PFE on phosphorylation of JNK, P38 and Erk1/2 in BEAS-2B cells in response to DP2 were investigated. As shown in Figure 4, comparing to GST treatment, DP2 significantly enhanced phosphorylation of JNK, P38 and Erk1/2 to 2.42, 4.21, and 2.48-fold of GST alone, respectively. the DP2-induced phosphorylation of JNK and P38 were diminished upon PFE pretreatment in a dose-dependent manner but not Erk1/2. With pretreatment of PFE at 50 µg/mL, levels of p-JNK, p-P38 and p-Erk1/2 were reduced to 1.18, 1.25 and 1.01-fold of GST alone. In addition, treated with GST (20 µg/mL) alone insignificantly affected phosphorylation of JNK, P38 and Erk1/2 as compared to negative control (DMSO).

### PFE suppressed degradation of IκBα and nuclear translocation of NF-κB in DP2-stimulated BEAS-2B cells

**Figure 5:**
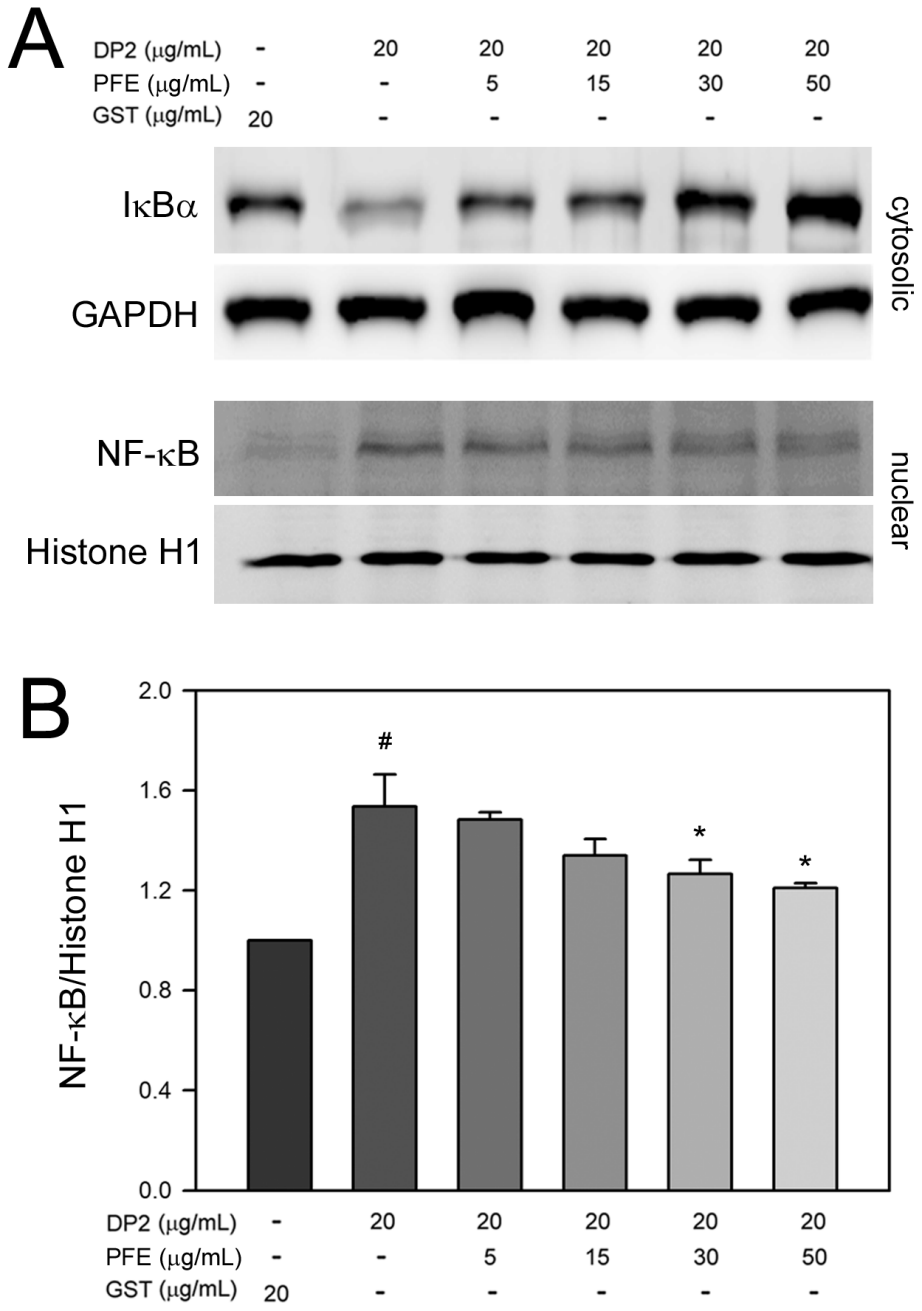
PFE elevated cytosolic IκBα and diminished nuclear NF-κB in DP2-stimulated BEAS-2B cells. After 16-starvation, cells were pretreated with 5, 15, 30 and 50 µg/mL PFE for 1 h, and then stimulated with 20 µg/mL DP2 for 30 min. The treated cells were lysed for (A) determination of cytosolic IκBα, or (B) distribution of cytosolic and nuclear NF-κB by immunoblot using specific antibodies and chemiluminescence development. Quantitative data was performed by densitometric analysis and obtained from three independent experiments. Level of GAPDH and histone H1 was used as cytosolic and nuclear control, respectively. #, *p*<0.05 as compared to GST alone; *, *P*<0.05 as compared to DP2 alone.

Transcription factor NF-κB plays a pivotal role in regulation of pro-inflammatory cytokines, and its nuclear translocation is associated with the expression of the pro-inflammatory cytokines. Thus, effects of PFE on degradation of IκBα and nuclear translocation of NF-κB in BEAS-2B cells exposed to DP2 were subsequently investigated. As shown in Figure 5A, exposure of BEAS-2B cells to DP2 led to a significant decrease of cytosolic IκBα level, the NF-κB inhibitor, contributing to nuclear translocation of NF-κB. In addition, PFE pretreatment restored the DP2-decreased cytosolic IκBαlevel and diminished nuclear NF-κB level increased by DP2 in a dose-dependent fashion. Further quantitative analysis showed that DP2 treatment increased level of nuclear NF-κB to 1.54±0.15-fold of GST alone (*P*<0.05), and PFE pretreatment (50 µg/mL) following DP2 treatment decreased nuclear NF-κB level to 1.21±0.03-fold of GST control (Figure 5B). These findings revealed that PFE inhibited not only degradation of IκBαbut also nuclear translocation of NF-κB.

## Discussion

Previous studies have shown that different extractions of *Perilla frutescens* exert various beneficial effects on airway inflammatory and hypersensitive disorders [19], [20]. Oh et al. have reported an ethanol extract of *Perilla frutescens* leaf and rosmarinic acid, the major component of the extract, is able to reduce expression of IL-1β, IL-6 and TNF-α in the nasal mucosa tissue of ovalbumin-sensitized mice [19]. Consistently, our results showed that PFE diminished the increased mRNA expression of both pro-allergic and pro-inflammatory cytokines in human bronchial epithelial cell BEAS-2B caused by DP2. In addition, our cytotoxicity analysis demonstrated that PFE up to 100 µg/mL insignificantly affected viability of BEAS-2B cell. These findings indicate that PFE is potential to protect airway epithelium from hypersensitiveness and inflammation evoked by pathogens.

Cytokines are critical in allergic intercellular networks, and they contribute to disease pathology through the recruitment and activation of pro-inflammatory leukocytes and in chronic disease to pro-fibrotic/remodeling events. T helper 2 (Th2) cytokines, including IL-4, IL-5 and IL-13, predominate primarily in mild to moderate allergic asthma. IL-4 and IL-13 are key Th2 cytokines that direct many of the important features of airway inflammation and remodeling in patients with allergic asthma. Thus, IL-4/IL-13/signal transducer and activator of transcription 6 (STAT6) pathway becomes promising targets for asthma therapies by using IL-4 receptor antagonists and anti-IL-13 mAbs [21]. GM-CSF released from bronchial epithelial cells of asthmatic patients can prolong the survival of eosinophils and enhance the release of mediators from those cells, contributing to the pathogenesis of airway hypersensitiveness [22], [23]. Our results demonstrated that PFE significantly reduced the enhanced mRNA expression and protein production of IL-4, IL-5, IL-13 and GM-CSF in BEAS-2B cell with exposure to DP2, indicating that PFE may attenuate the pro-allergic Th2 responses triggered by airway epithelium in response to aeroallergen.

The mucosal epithelium itself is responsible for the synthesis and release of cytokines that cause the selective recruitment, retention, and accumulation of lymphocytes and leukocytes [24]. IL-6 and IL-8 are two classic pro-inflammatory cytokines that play important roles in bronchial epithelial function [25], [26]. IL-6 is highly elevated cytokine in mouse model of chronic obstructive pulmonary disease (COPD)-like inflammation [27] and has been implicated in inflammatory responses in human COPD [28], [29]. IL-8 is a CXC chemokine that is recognized as a potent effector of neutrophil functions [30]. IL-8 specifically attracts several cell types involved in inflammation. After NF-κB activation by TLR signaling, IL-8 expression increases [31] and this cytokine has been implicated as a causative agent in a broad range of pathological conditions including rhinitis, bronchitis, and bacterial infections [32], [33]. CC-chemokines such as CCL2/MCP-1, which are chemotactic for mononuclear phagocytes, were identified as pro-fibrotic mediators. Subsequent studies with CCR2-deficient mice produced similar results, confirming critical roles for CCL2-mediated signaling pathways in fibrogenesis [34], [35]. In addition, CCL2 production by epithelial cells and macrophages has been reported to contribute to rhinovirus-induced airway hyperresponsiveness and inflammation in a mouse model of allergic airways disease and may play a role in rhinovirus-induced asthma exacerbations [36]. Our findings revealed that PFE significantly reduced the elevated mRNA expression and protein production of IL-6, IL-8 and MCP-1 in BEAS-2B cell exposed to DP2, suggesting that PFE may suppress the inflammatory responses of airway epithelium induced by aeroallergen.

Since MAPKs such as Erk1/2, JNK and P38 are involved in activation and upregulation of NF-κB, and the consequent induction of pro-inflammatory mediators [37], intervention of these signaling pathways could therefore be considered as a possible therapeutic target for the development of anti-inflammatory agents [38]. Moreover, the involvement of P38 activation in the production of IL-6 [39], [40] and IL-8 [41] has been previously reported in airway epithelial cells. Activation of NF-κB is considered to be a crucial inflammatory nuclear transcription factor and a central mediator of inflammatory responses [42]. It has been demonstrated that expression of pro-allergic and pro-inflammatory cytokines in airway epithelia can be regulated via JNK/NF-κB- [43]-[45] or P38/NF-κB- [40], [41] signaling cascades. In the present study, we also found that enhanced nuclear translocation of NF-κB and the concomitant activation of JNK and P38 was suppressed by PFE pretreatment in BEAS-2B cells exposed to DP2. These findings indicate that PFE effectively inhibited DP2-induced mRNA expression of pro-allergic and pro-inflammatory cytokines, which may attribute/be attributed to inhibition of the JNK/P38 and the consequent suppression of NF-κB nuclear translocation.

In conclusion, our results show that major allergen DP2 induces both gene expression and protein production of pro-allergic and pro-inflammatory cytokines. The increases in production of pro-allergic and pro-inflammatory cytokines are significantly suppressed by PFE. Accordingly, it is suggested that PFE should be beneficial to ameliorate inflammation and hypersensitiveness of airway epithelium in response to aeroallergens or inhalant pathogens.
